# Educational innovation in cardiovascular care: Developing a curriculum for women's cardiovascular health^☆^

**DOI:** 10.1016/j.ahjo.2025.100693

**Published:** 2025-12-09

**Authors:** Giorgia Borgarelli, Cristiane C. Singulane, Paul Montana, Lucie Lefbom, Hakinya Karra, Kelsey Watts, Kara Harrison, Jennifer M. Choffel, Christopher S. Ennen, Aditya M. Sharma, Kelly E. Wingerter, Victor Soukoulis, Patricia F. Rodriguez-Lozano

**Affiliations:** a Department of Family Medicine, University of Virginia Health, Charlottesville, VA, USA; b Department of Medicine, Cardiovascular Division, University of Virginia Health, Charlottesville, VA, USA; c Department of Medicine, University of Virginia Health, Charlottesville, VA, USA; d Center for Public Health Genomics, University of Virginia School of Medicine, Charlottesville, VA, USA; e Department of Obstetrics and Gynecology, University of Virginia School of Medicine, Charlottesville, VA, USA; f Department of Radiology and Medical Imaging, University of Virginia Health, Charlottesville, VA, USA

**Keywords:** Women's Health, Multidisciplinary Care, Cardio-obstetrics, Innovation on education, Health disparities

## Abstract

Cardiovascular disease (CVD) is the leading cause of death among women in the United States, with significant knowledge gaps in sex-specific care among providers. To address this, we developed and implemented a structured, multidisciplinary women's cardiovascular health (CVH) curriculum within the cardiovascular fellowship program at the University of Virginia. The curriculum focused on women-specific cardiovascular health, including cardio-obstetrics, spontaneous coronary artery dissection (SCAD) and coronary microvascular disease (CMD), and encompassed didactic lectures and unique experiential learning through clinical work, research, and community outreach. This multidisciplinary approach enhances individualized care and prepares trainees to recognize and treat the unique cardiovascular needs of women. Continued development of such curricula is crucial for reducing morbidity and mortality disparities in CVD.

## 1 Introduction

Cardiovascular disease (CVD) remains the leading cause of death among women in the United States, accounting for over 400,000 deaths per year. Despite efforts, there has been a lower reduction in mortality over time in women compared to men (1). Alarming trends show more young women hospitalized with myocardial infarction (MI), yet they are less likely to receive guideline-based treatment (2). It is now recognized that women have a unique phenotype of heart disease with differences in symptoms, sex-specific risk factors, and underlying conditions (3). Moreover, the United States has the highest maternal mortality in the developed world, with CVD as the leading cause of pregnancy-related death (4). Although awareness and research on women's cardiovascular health (CVH) have improved in recent years, significant knowledge gaps persist among cardiovascular professionals. Current physician trainees endorse a lack of and a need for comprehensive clinical training in Sex and Gender Medicine (5). A contributing factor to this issue is the lack of standardized sex and gender-specific medicine curricula offered to medical trainees.

Emerging formal programs show promising improvement in knowledge on this important topic ([6], [7], [8]), however only a few exist, and the learning is primarily didactic. Consequently, trainees have limited opportunity for clinical application to solidify their knowledge base. We aimed to address these gaps by developing an innovative curriculum emphasizing intensive and widespread experiential learning.

We piloted a single-center experience, introducing a structured, multidisciplinary women's CVH curriculum within our cardiovascular fellowship. We structured our curriculum around the principles of The University of Virginia (UVA) Women's Heart Health Program (WHHP), which offers specialized care through several multidisciplinary clinics. Our curriculum offered formal teaching by diverse team members and emphasized collaboration, echoing the emerging idea of a “heart team” model. A distinctive feature was the substantial experiential portion, which integrated advanced cardiac imaging tailored for women's cardiovascular needs, collaboration across multidisciplinary clinics focusing on spontaneous coronary artery dissection (SCAD), fibromuscular dysplasia (FMD), cardio-obstetrics (OB), menopause-related care, research involvement, and community outreach. Engaging fellows in all these sectors ensured comprehensive insights into women's CVD and addressed disparities in care and outcomes.

## 2 UVA's women's heart health program

The WHHP at UVA provides personalized and comprehensive cardiovascular care tailored to women at every stage of their lives. The program aims to improve education for women and healthcare providers, expand research, and reduce social barriers to cardiovascular care. This initiative promotes collaboration among healthcare disciplines that provide care for women, from adulthood to middle age to geriatric. The WHHP is structured into three sections: the Women's Clinic, the SCAD/FMD Clinic, and the Cardio-OB Clinic.

• **Women's Clinic**: This clinic occurs twice weekly and focuses on screening and primary prevention, as well as managing women with coronary artery disease (CAD). It addresses the menopause transition and provides risk stratification for use of hormone replacement therapy. Additionally, the clinic specializes in coronary microvascular disease (CMD) and MI with non-obstructive coronary arteries (MINOCA).
• **SCAD/FMD Clinic**: As a referral center, this clinic collaborates with vascular medicine to manage SCAD and its association with FMD. This clinic occurs once weekly.
• **Cardio-OB Clinic**: This section incorporates maternal-fetal medicine (MFM) to manage high-risk pregnancies and postpartum cardiovascular care. It provides specialized care for pregnant women with cardiovascular risk factors and addresses early cardiovascular risk in the postpartum period. The general cardio-OB clinic occurs weekly, while the combined MFM/cardio-OB clinic occurs twice monthly.

Patients begin with a comprehensive assessment at the Women's Clinic by a core team including a pharmacist, social worker, cardiologist, and advanced practice provider. They are screened for social and psychological factors, such as depression, anxiety, and post-traumatic stress disorder, and appropriate referrals are made.

When additional expertise is required, patients are referred to the ancillary team, including psychologists, nutritionists, and exercise physiologists. Targeted interventions tailored to the patient's needs are then developed. Follow-up appointments with ancillary specialists may be conducted in-person or via telehealth, ensuring continuity of care and accessibility.

To further refine care, patients are screened using tools embedded within the electronic medical record system to identify sex-specific and under-recognized cardiovascular risk factors. These include psychological, social, economic, and cultural influences disproportionately affecting women, such as intimate partner violence, socioeconomic challenges, and sociocultural roles. Additionally, conditions unique to women—such as early menopause, preterm delivery, gestational hypertensive disorders, gestational diabetes, and polycystic ovary syndrome—are assessed for their potential long-term impact on cardiovascular risk, enabling tailored preventive strategies.

**Fig. 1:**
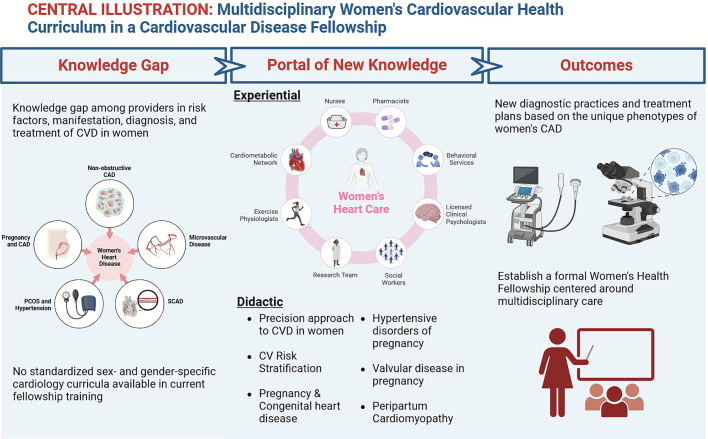
– Central illustration. Knowledge gaps on the unique factors of women's heart disease were addressed in our multidisciplinary didactic and experiential curriculum, with the aim to improve diagnostic practices and treatment plans in women. CAD = coronary artery disease; SCAD = spontaneous coronary artery dissection; CVD = cardiovascular disease; PCOS = polycystic ovary syndrome.

This multidisciplinary model ensures that patients receive comprehensive, holistic care, addressing cardiovascular disease from multiple perspectives. Simultaneously, it provides trainees with invaluable experiential learning opportunities, enabling them to integrate didactic knowledge with real-world practice and enhance their ability to manage women's cardiovascular health effectively **(see Central Illustration.** Fig. 1**).**

## 3 Approach to curriculum design

We aimed to provide a broad and robust overview of the presentation, diagnosis, and management of CVD in women. Our curriculum combined didactic and experiential learning, leveraging UVA's unique WHHP to ensure multidisciplinary teaching. The innovative individualized care offered at the WHHP extends into the physician educators delivering the didactic portion of the curriculum. Institutional experts included cardio-obstetricians, heart failure specialists, pediatric congenital cardiologists, cardiac imagers, vascular medicine specialists, and hematologists. Each delivered lectures in their area of expertise within the context of women's CVD.

Additionally, through the experiential component of the program, trainees rotated through all the different clinics, learning from specialized nurses, pharmacists, exercise physiologists, research teams, social workers, psychologists, and behavioral scientists. Training in this setting offered trainees first-hand experience in personalized multidisciplinary care for women. The elective rotation also included dedicated time for research and advanced imaging (computed tomography [CT] / cardiac magnetic resonance [CMR]) to learn about evaluation of heart disease in women. A wide range of research topics were available for trainees to explore and expand on, given institutional access to SCAD/FMD registry, cardio-OB registry, and clinical trials in innovative therapies on CMD. Further, through the clinic's frequent involvement in the community, trainees participated in outreach initiatives, such as cardiovascular screenings and educational workshops targeting underserved populations.

## 4 Curriculum development and implementation: content, objectives, and educational strategies

**Fig. 2:**
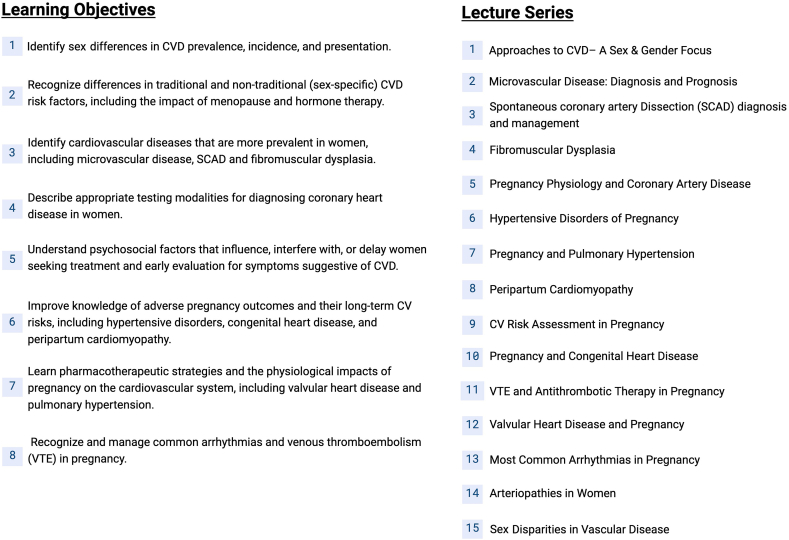
Outline of established learning objectives and lecture series delivered to fellows in training.

**Fig. 3:**
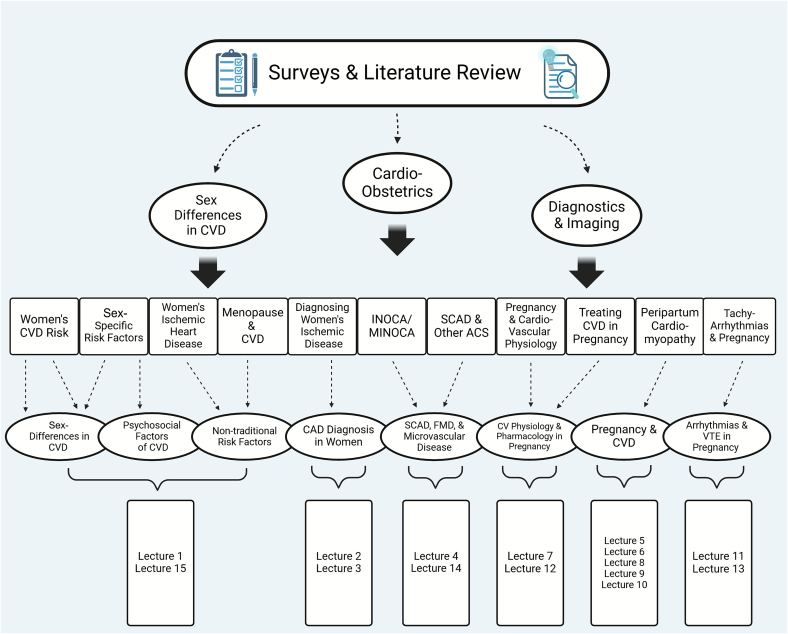
Three primary knowledge gaps in women's cardiovascular health were determined based on institutional surveys and literature review. Competencies were carefully crafted to address these gaps, which were subsequently condensed into eight tangible learning objectives. Finally, focused lectures were developed to cover the material of the learning objectives. Refer to Fig. 2 for corresponding lecture titles.

We began by assessing the existing gaps in knowledge through institutional surveys and literature review (4,5,[8], [9], [10]). We determined the largest deficiencies were in sex-specific differences in CVD, cardio-OB, and imaging training. We thus developed competencies that prioritized these topics, while also providing comprehensive understanding of women's CVH. From these, we carefully crafted learning objectives that addressed the unmet needs of trainee knowledge of women's CVH. The mapping process for the didactic portion of the curriculum ensured alignment of lecture topics with survey data and clinical objectives. Institutional experts delivered one-hour lectures in their area of expertise based on the learning objectives. The didactic sessions included 15 lectures, which are summarized along with the curriculum learning objectives in Fig. 2**.** The lectures were dispersed throughout the length of fellowship training and attendance was mandatory (Fig. 3)**.**

The experiential portion of the curriculum is unique to our design and relied on the state-of-the-art Women's Heart Disease Clinic to provide trainees varied experiences in specialized areas of women's CVH. The elective experiential portion included a week-long clinical rotation, research involvement in various projects, and participation in community outreach over one month. During the clinical rotation, trainees divided their time among the Women's Heart, Cardio-Ob, and SCAD-FMD clinics, spending one-half to one day in each clinic. One day of the rotation was dedicated entirely to reviewing women's cardiac imaging with in-house specialists, another novel aspect of our curriculum. For the research component, trainees had access to extensive projects and registries, including our SCAD/FMD registry, OB registry, and clinical trials in microvascular disease. Trainees were supported in crafting their own projects or joining existing ones, and their involvement could extend beyond the elective. Finally, as part of the elective, trainees attended 4 community events, including educational workshops and screening fairs. The experiential portion of the curriculum extended over one month and allowed trainees to practice first-hand the knowledge they gained throughout the didactic portion of the curriculum. Importantly, they gained extensive multidisciplinary knowledge on women's cardiovascular health due to the robust and unique network offered by the WHHP.

The curriculum has received positive reception among trainees and faculty**.** Trainees highlighted that the curriculum aligned with their learning needs and would directly benefit their future clinical practice and patient outcomes. Suggestions for improvement included offering the curriculum more frequently, expanding training on medication safety in pregnancy, and providing additional refreshers on pregnancy-related cardiovascular physiology.

## 5 Conclusion

Establishing a formal curriculum in women's CVH is essential to close the existing knowledge gap among providers and trainees, promote standardized care, and ultimately improve patient outcomes. Our multidisciplinary model—engaging specialists across medical, behavioral, and social fields—prepares trainees to provide holistic, individualized care. Through hands-on multidisciplinary clinics, imaging training and dedicated research time, the curriculum strengthens clinical skills and research engagement. The community outreach initiatives unique to our curriculum are a key component, teaching trainees the importance of public health education and early detection of CVD in underserved populations, addressing healthcare disparities. This comprehensive, patient-centered approach will hopefully lead to better clinical outcomes and a reduction in the disparities in CVD morbidity and mortality between women and men.

## CRediT authorship contribution statement

**Giorgia Borgarelli:** Writing – review & editing, Writing – original draft, Visualization. **Cristiane C. Singulane:** Writing – original draft, Writing – review & editing, Visualization. **Paul Montana:** Methodology, Investigation, Data curation. **Lucie Lefbom:** Writing – review & editing, Writing – original draft. **Hakinya Karra:** Writing – review & editing, Writing – original draft, Visualization. **Kelsey Watts:** Visualization. **Kara Harrison:** Writing – review & editing, Methodology, Conceptualization. **Jennifer M. Choffel:** Writing – review & editing, Project administration. **Christopher S. Ennen:** Writing – review & editing. **Aditya M. Sharma:** Writing – review & editing. **Kelly E. Wingerter:** Writing – review & editing. **Victor Soukoulis:** Writing – review & editing, Methodology, Investigation, Conceptualization. **Patricia F. Rodriguez-Lozano:** Writing – review & editing, Writing – original draft, Validation, Supervision, Resources, Project administration, Methodology, Formal analysis, Conceptualization.

## Animal welfare

This research did not involve the use of animals.

## Informed consent

Written informed consent was obtained for anonymized patient information to be published in this article.

## Ethics approval

Ethical approval for this study/case/case series was obtained from the Institutional Review Board for Social & Behavioral Sciences.

## Funding

This research received no specific grant from any funding agency in the public, commercial, or not-for-profit sectors.

## Declaration of competing interest

The authors declare that they have no known competing financial interests or personal relationships that could have appeared to influence the work reported in this paper.
